# ‘It would be much easier if we were just quiet and disappeared’: Parents silenced in the experience of caring for children with rare diseases

**DOI:** 10.1111/hex.12958

**Published:** 2019-08-29

**Authors:** Genevieve Currie, Joanna Szabo

**Affiliations:** ^1^ School of Nursing and Midwifery, Faculty of Health, Community and Education Mount Royal University Calgary Alberta Canada

**Keywords:** caregivers, children, chronic disease, health‐care system, neurodevelopmental disease, parents, rare disease, social care supports

## Abstract

**Background:**

Parent experiences of caring for children with neurodevelopmental disease have been silenced and constrained by social, political and health influences. There is a need to co‐construct new meanings and interpretations of parenting a child with complex disabilities by having an increased understanding of the struggles and barriers for parents.

**Methods:**

A hermeneutic phenomenology approach was applied in this inquiry. Fifteen parents of children with rare neurodevelopmental diseases participated in semi‐structured interviews.

**Results:**

Parents experienced silencing or being silenced within interactions with health‐care and social care systems and providers. Interpretive thematic analysis revealed three insights: (a) parents experience a sense of disconnect and silencing as little is known or understood by health‐care providers about the experience of caring for children at home; (b) parents make strong efforts to be heard and acquire services within health and social systems as fighters, saviours and navigators; and (c) parents sacrifice themselves to the caregiving role and become therapists and caregivers to their medically fragile children at the cost of losing themselves as parents.

**Conclusion:**

An understanding of parents’ experiences in caring for a child with a rare neurodevelopmental disease may provide insight to systemic health and social support challenges faced by families and mitigate appropriate and supportive policies and services.

## 1 INTRODUCTION

Rare neurodevelopmental diseases (NDD) pose significant health and social challenges; however, there is insufficient understanding amongst health‐care and social care providers about parents’ experience of caring for children with these diseases. Revealing the experiences of parenting children with rare diseases exposes narratives of parents struggling in meeting their children's complex health‐care and social needs. Parental narratives have been unvoiced, misunderstood and incoherent to health‐care providers (HcPs) and the community at large, often leading to the silencing of these multivocal experiences.^1^, ^2^ Unsilencing and illuminating parent voices and experiences can increase insights into, and understanding of the challenges of living with complex disabilities. Providing this ‘insider view’ may cultivate health‐care and social care practices and policies that are informed by understanding actual experiences, perceptions of parents, and parents’ own personal and practical knowledge 3 when caring for children with NDDs.

## 2 BACKGROUND

In this literature review, the first section addresses rare diseases, specifically NDDs; the second section explores parents’ experience in caring for children with complex diseases; and the third section describes social constructs of parenting, particularly constructs influencing expression of parent care experiences. Together, these provide some of the main considerations that shape this proposed inquiry.

Rare diseases disproportionately impact more children than adults and can lead to premature death^4^; 80% of rare diseases are caused by genetic changes.^4^ Contained within the rare disease classification are genetic NDDs that influence the development and function of the brain and other body systems due to mutation in a single gene or multiple genes.^5^ Many rare NDDs cause substantial disabilities for children in multiple arenas with cognitive impairment, growth and development delays, physical disabilities, severe neurobehavioural issues and social impairments.^6^ The burden of care placed on parents is significant in meeting these complex challenges,^7^ and these experiences have been underexplored by health systems and providers.^1^, ^2^, ^8^, ^9^

Narratives from parents with children with disabilities have remained at the edge of larger medical and social systems, potentially contributing to parental needs being taken for granted and remaining unaddressed.^10^, ^11^, ^12^ Parents must become expert care providers addressing pervasive health and social needs, navigating fragmented discoordinated care within health‐care and government support systems, while carrying a heavy burden of care.^13^ Within the finite studies of parent experiences in caring for children with medical complexity, parents describe carrying significant emotional and social responsibility when caring for their children because of overwhelming circumstances and struggles.^13^, ^14^, ^15^, ^16^ Parents express being stressed, overwhelmed and overextended with providing care at home where similar care within hospital settings required highly specialized providers.^11^, ^17^

Even with intensive care needs, parents do not receive the supports and services they require to meet their children's complex care needs.^12^, ^17^ This lack of responsiveness may be related to social, historical, cultural and medical expectations of parents and resulting social narratives.^18^, ^19^ In particular, parental experiences of caring for children with rare complex diseases do not resonate within socially constructed discourses of parenthood and thus remain unstoried.^11^, ^19^ The social constructs of embodied mother, ‘good mother’, and resilience associated with disabilities may possibly contribute to suppressing and diminishing parent experiences. Mothers typically are more responsible for managing the daily medical and social care needs of children with rare diseases; therefore, literature focusing on social expectations for mothering is important to include here.^7^

The concept of the ‘embodied mother’ merges the experience of the disabled child with the mother's physical and social body as the mother navigates through social structures.^20^ Mothers become part of their children's intimate vulnerable spaces and are indeed silenced. In this way, the mother too can become disabled as ‘witness and participant’^20^ experiencing the public landscape of the disabled child. Glenn also speaks to social silencing as a power discourse within patriarchal systems that disempowers mother and parent voices.^21^ Carpenter and Austin extend this understanding, stating mothers’ experiences have been unvoiced and unstoried by HcPs when their identities and personhood become fused with those of their children as they struggle to meet their children's health and social needs.^11^ This muting contributes to mothers feeling diminished and ‘disabled’ as parents and caregivers.^11^

Another social construct within the literature affecting care providers’ response to parents, primarily mothers, includes ideologies of the ‘good mother’.^22^ Dominant constructs that focus on the mother within the family create and sustain unrealistic expectations for parenting behaviours. Good mothers are nurturing, and selfless while providing unconditional love^22^; are devoted to their children; and seek needed resources for their children.^23^, ^24^ The literature reveals there are even higher expectations within society for mothers caring for children with disabilities. Mothers are to be selfless, self‐sacrificing, emotionally compassionate, patient, resilient, resourceful, self‐directed, problem solvers, find meaning in difficult situations, and be hyperresponsive and hyper‐responsible.^19^, ^25^, ^26^

Elaborating further on parents’ experience with caring for children with complex care needs, researchers historically display loss and grief as an overwhelming experience with little recovery. Studies are grounded in loss of the ‘typical’ child.^27^ Recently, authors have shifted to parental models of acceptance and resilience in the face of adversity.^28^, ^29^ This concept of resilience suggests adaptation, transformation, and eventual growth and transcendence for families.^30^, ^31^ The literature suggests an expectation of recovery and moving on from difficult life circumstances and struggle.

In summary, these social constructs may not provide insight into the experience of caring for children who require intensive intervention and management. Complex, struggling or overwhelming parenting experiences are not always coherent or recognized within these social constructs, and parent experiences may be constrained and silenced by larger influences and thus remain ‘unstoried’. There is a need to expand understanding by constructing new meanings and interpretations of parents’ experiences of caring for a child with a rare NDD. Parent narratives about caring for children with NDDs can offer deeper and richer insights into parents’ experiences than exist in the current literature. This knowledge may inform the development of sensitive, supportive, appropriate care strategies, practices and policies. The research explored the following question: What are parents' perceptions and experiences of support from health and social service communities when living with a child with a rare neurodevelopmental disease (NDD)? 

## 3 METHODS

### 3.1 Methodology

Hermeneutic phenomenological inquiry was chosen for this research study as its philosophical beliefs increase understanding of what has been silenced or unstoried within the parent experience.^32^ Within hermeneutic phenomenology, narratives are constructed to honour the historical, cultural, political, relational and contextual understandings of experiences and the generative and additive effects of individual or social influences.^33^, ^34^ The goal of hermeneutic phenomenology was to understand the meanings associated with parents’ everyday experiences in caring for children with rare NDDs.

### 3.2 Inclusion criteria

Eligibility for the study involved meeting the definition of a rare disease as set by Rare Disease Foundation of Canada with parents whose children had a rare NDD (defined as having a prevalence of below 1 in 2000 live births).^35^

### 3.3 Recruitment

Fifteen parents (11 mothers and 4 fathers) were recruited for the study using purposive sampling.^36^ Parents were recruited from medical genetic, endocrine, and neuropsychiatry clinics and self‐referrals from parent support groups within several Western Canadian hospitals. While the sample size was small, it was typical for this type of inquiry.^37^ Adequacy of the sample was achieved when the interpretation expanded understanding.^38^

### 3.4 Participants

Parents were 30‐45 years of age with children 11 years of age and younger, diagnosed with rare NDDs within 2 years of birth. Parents had high school education with the majority having several years of post‐secondary education. Fourteen of the parents were living with a partner; one was a single parent because of divorce. Four of the participants were married to each other and were interviewed separately. Parents had middle to higher income status, with nine mothers and all fathers employed in either part time or full‐time employment. Five of the parents lived in rural settings. See Table 1: Participant Demographics.

**Table 1:** Participant characteristics

Gender	Age	Marital status	Education	Employment status	Yearly household income
Female	32	Married/Domestic Partnership	Some College	Part time	$50 000–$74 999
Female	30	Married/Domestic Partnership	Some College	Stay at home parent	$50 000–$74 999
Male	33	Married/Domestic Partnership	College	Full time	$50 000–$74 999
Female	40	Divorced/Separated	University (Bachelor's)	Part time	$50 000–$74 999
Male	35	Married/Domestic Partnership	College	Full time	$50 000–$74 999
Female	35	Married/Domestic Partnership	College	Full time	$50 000–$74 999
Female	39	Married/Domestic Partnership	College	Full time	$100 000–$149 999
Female	32	Married/Domestic Partnership	Some College	Stay at home parent	$50 000–$74 999
Female	34	Married/Domestic Partnership	University (Bachelor's)	Full time	$75 000‐$99 999
Female	38	Married/Domestic Partnership	College	Full time	$75 000‐$99 999
Female	43	Married/Domestic Partnership	College	Full time	$75 000‐$99 999
Female	44	Married/Domestic Partnership	University (Bachelor's)	Full time	$75 000‐$99 999
Male	37	Married/Domestic Partnership	College	Full time	$100 000–$149 999
Male	45	Married/Domestic Partnership	University (Master's)	Full time	$100 000–$149 999
Female	42	Married/Domestic Partnership	University (Master's)	Full time	$100 000–$149 999

Note

No detailed demographic information has been provided, so as to protect the personal identity of the participants.

### 3.5 Data collection

Interviews were conducted from June 2016 to November 2016 in two urban centres as well as several rural areas in Western Canada. The setting for the interviews was negotiated with the participants. One participant chose to be interviewed at a private work setting; all other participants elected to be interviewed in their own homes. Prior to commencing the semi‐structured interviews, human research ethics approval was received for this research project. Due to the sensitive nature of the narratives, the researchers worked closely with participants to attend vulnerability with disclosure. In‐depth, face‐to‐face interviews lasted from 45 to 120 minutes to promote engagement with participants and provide ample opportunity for parents to describe their perceptions. All interviews were recorded and transcribed verbatim. Researchers checked the transcripts for accuracy before analysis.

### 3.6 Data analysis and rigour

Researchers read, reflected, interpreted and re‐read the data (interviews transcripts and interpretive notes) individually as well as collectively multiple times^39^ to bring forth impressions, alternative explanations, divergent patterns, and insights from the narratives and interpretive notes to generate increased understanding.^40^, ^41^ The use of questions, reflections and understanding through interpretations revealed complexities caring for children with rare NDDs. Insights were constructed to uncover the parents’ experience.^39^

## 4 FINDINGS

We explored parents’ daily experiences of caring for children with complex care needs. Three insights emerged and provide new interpretations of the parents’ experience of parenting a child with a rare NDD. These insights were themed: (a) disconnect, (b) in the ring and (c) self‐sacrifice. Additional insights considering the parent experience are found elsewhere.^42^ Participant pseudonyms are used to ensure participant anonymity and to specify the gender of the participant.

### 4.1 Disconnect: ‘…And They Talk to You Like They Have Never Met You’

Parents shared the experience of caring for children with rare diseases has rarely been sought by HcPs. This lack of exploration has led to a general disconnect amongst HcPs about the disease and its manifestations for families: ‘Trying to get people [HcPs] to understand what he has is really difficult. Nobody knows about it [the disease]. We have to find our own help’ (Alexa). One parent described the emotional impact of disconnected care: ‘The amount of times we have gone to emergency and they talk to you like they've never met you…It's fatiguing, emotionally draining and almost insulting’ (Kim).

Parents described depersonalization within compartmentalized care when they had to tell their story with each provider: ‘We have told our story so many times that it doesn't seem like our story anymore. It takes the personalization out of it’ (Wendy). Another parent expanded on the sense of detachment within health‐care practices that treat repeated contacts with families as disconnected entities:

‘One of the things that drives me crazy is when you go to these appointments and you think you’ve already filled out the same things. Don’t you know this already? Then you have to dig in your brain and write it out and get stressed. Then you go to the next step and see the person and they ask you all the questions again.… There must be some rhyme or reason to it, but it seems like the most tedious, stupid thing, and it makes you feel like no one is listening or cares’. (Kim)

Adele described the disconnect and lack of integration within subspecialty care with a child with comorbidities:

All of the clinics at the hospital seemed very disjointed. You are going to the GI [gastrointestinal] clinic, but GI clinic has no clue what cardio [cardiology] clinic is doing. You really had to be on top of it and organised to make sure you asked the right questions. (Adele)

One father communicated how his efforts in caring for his child were not recognized by a HcP:

A couple of months ago there was a report from the doctor, and he said it’s a miracle she’s doing so well. J and I were close to strangling him. It’s not a miracle. It’s two people working at the edge of their scientific, emotional, and parenting capabilities all at once, doing what drugs can’t do. (Josh)

Health‐care providers remained detached and disconnected from parents and their caring experiences when they did not incorporate parental expertise into plans of care. Maya described how her physician reacted when she brought forward information about her child's medical condition: ‘Sometimes I find that they [physicians] don't really believe me. They think they know more and can be condescending’. This same parent described a lack of collaboration and not feeling supported to share her evidence‐based knowledge: ‘Some of the unhelpful things are when they [HCPs] are not receptive to you bringing your research to the table. They get annoyed when you go on websites’. And another parent expanded on this lack of connection to parents’ knowledge of their children: ‘We keep giving them information and it doesn't matter. They don't incorporate that information into treatment plans or protocols’ (Talia).

In summary, parents described a disconnect from systems and providers when seeking supports and resources. There was a perception that delivery models contributed to the disconnection between HcPs and parents regarding experiences of caring for children with rare NDDs.

### 4.2 In the Ring: ‘I’m the Parent There Every Day Fighting for my Child’

Parents amplified their voices and fought back when their experiences were not heard within health‐care and social care systems. One parent stated, ‘I’m the parent there every day fighting for my child’ (Jenna). It took voices of substance to navigate through powerful hierarchical systems and structures with a patchwork of supports and resources to meet chronic care needs: ‘It feels like a fight every day. We fight for her. We fight the system, and we fight for any support we can actually get. It's very isolating and very lonely and very frustrating.… It's terribly soul crushing’ (Talia). Similarly, another parent noted, ‘Everything that has happened since then, has all been stuff that I really clawed for’ (Adele).

Within the parent role, there are expectations to stand up for your child, but parents became frustrated with the need to fight for essential care needs: ‘We are the people who ask tons of questions and occasionally complain about behavior. Otherwise known as “fighting for your kids because no one else will”’ (Josh). Another parent said, ‘You have to get into the mama bear mode when it isn't necessarily natural when you are trying to be nurturing and caring. Then you have to switch over and take on a more forceful role’ (Maya). The same parent exposed a strong effort to get services: ‘We have knocked on doors of offices and were told we weren't invited, that we needed an appointment. Sat in emergency and told to go home but we said no and sat there until we saw someone. It's exhausting and takes lots of time and effort’ (Maya).

When a parent was asked if anyone from the health‐care team listened to her, she replied with conviction, ‘Yes, because I’ve made myself heard. I don't think that they listen, but I kept shouting, being that annoying person who continuously calls.… We follow up and send emails and make phone calls and we don't get missed anymore’ (Maya). Another parent said, ‘They're calling for a neurology consult, and nobody showed up. I began screaming and said, ‘Does it take brain damage before a neurologist shows up?’ We got a neurologist that day to show up’ (Josh). These narratives spoke to the necessity to be heard.

Parents relayed stories of pushing back against ineffective care models and social systems as fighters, saviours and navigators for their children. One participant, when asked about her challenges with addressing care needs, stated, ‘I can't imagine not fighting for my child, because if I don't fight for him, who is going to? I can't imagine how you would navigate that [health care] system. Even for me to navigate it and I’m home. There are days where you want to pull your hair out’ (Jenna). Strong metaphors were used to describe the struggle to be heard: ‘We are like people in a war. Our consequences affect one life, not many. Our job is to keep her alive. It's the job of every parent to keep their child alive. The reality of what that means is a lot more visceral to us’ (Josh). Another father expressed discomfort in fighting for rights for his child: ‘The more you bitch, the more you get things done. People who let the system work get left behind. If I have to be an ass to get something done, then I’ll do it. It's not my nature to do that’ (Ken).

In short, there was a sense of pressure to manage the disease, navigate the health‐care and social care systems, and strongly advocate for supports to meet care needs. Parents described the struggle and effort to be heard while being ‘in the ring’ with health‐care and social care systems and structures.

### 4.3 Self‐Sacrifice: ‘You are Never Off Duty’

Parents revealed functioning within unrecognized roles as primary therapist and caregivers while addressing their children's care needs. This required self‐sacrifice on an hourly if not minute by minute basis. ‘I don't come from any medical background, and now I am the nurse and giving the meds, tubes, and suctioning. It almost compromises the mothering role, that you don't get to be the safe one when you have to be the one who is there for all of the painful things instead’ (Hannah). Parents divulged a sense of desperation that they were never off duty and it was up to them to manage their children's diseases. There was a sense of overwhelming accountability and responsibility if they did not do it correctly: ‘She can be as successful as she wants to be, but only if we can protect her, and we might fail’ (Josh). One father described the burden on parents: ‘You get those stupid articles that tell you about the wonderful parent that did twenty hours of therapy. I don't know what to do and I need to have a job’ (Ken).

Parents had to be resourceful and determine appropriate supports for their children: ‘We spend a ton of time researching and finding information’ (Jenna). Parents felt inadequate, and their efforts were not sufficient: ‘I feel like it is never enough. No matter what I do, there's always more that I could be doing. Someone else is doing it better’ (Sara). The same mother discussed an ineffective system relying on parents to meet care needs beyond most parents’ expertise:

I just want to be her Mom. We are expected to do things as parents and know things as parents that you as a typical parent wouldn’t have an expectation for medically. I’m not saying I don’t want to do anything to help her. I’ll support her, but I am no expert. (Sara)

Parents described the expectation to be hyperresponsive and hypervigilant when caring for their children: ‘There are so many things that are required.… There are so many have to's’ (Sara). Another parent noted, ‘It's not like these are one‐time events, they are a repetition.… It is like running on empty in a hypervigilant state’ (Kim). This places great responsibility on families: ‘We never go out because someone has to stay with her’ (Sara).

Parents also revealed the experience of seeking medical care and the mixed messages they received from HcPs. ‘They [the seizures] are scary. People don't get it and don't understand. We know between the two of us, whether it's calling 911, we get mixed feedback. ‘Well why didn't you bring them in sooner’ or ‘Why did you bring them in?’ We get a lot of that’ (Wendy). One father expressed how difficult it was to manage the medical fragility with his child:

You can have a perfectly normal healthy child, and you could lose your child. The difference in our case is that awareness that you can lose your child and the actuality of almost losing your child is much more intense and constant. I don’t know if we’re suffering from PTSD [posttraumatic stress disorder], but we haven’t slept for three years for more than a few hours. (Josh)

Hypervigilance led parents to uncover any medical and social supports while managing daily care needs: ‘It has been a lot of finding our own information, finding those answers and being overly cautious for everything that is happening’ (Wendy). This hypervigilance brought feelings of helplessness and vulnerability at great personal cost: ‘You are on all the time. I think the nature of parenting kids with special needs, you are so tired, and feel do much older than you are’ (Kim).

One of the sacrifices for parents was opening their homes to support workers on a daily basis. Although parents valued the support and help they were receiving for their children, they also expressed the invasion of privacy and loss of boundaries within private spheres: ‘I appreciate the help, but I don't want someone in my house’ (Wendy). Parents expressed lack of privateness from therapists and support workers: ‘You have no privacy.… As a parent you say or do something and wish you wouldn't have, and now you have another adult in there [your home] as well. I didn't feel judged by the aide, but I felt it wasn't fair to the other kids’ (Sara). Parents described the expectation they should feel privileged for the intrusion because they were receiving specialized care. ‘I think there's a lack of awareness and lack of understanding even from people close to us. They thought if you have nurses in your house everything should be wonderful; they don't know why you are complaining’ (Maya)*.* This same parent described the disruption and sense of judgement within her home:

As a new mom I found it hard not to feel judged when you’re having an odd day and want to plunk the toddler in front of the TV and there’s someone in your house. You feel the need to be on. You can’t feel frustrated and yell at the kid because there’s someone here. When you want to wear pajamas all day. When I want to nurse, I think I’ve nursed him in front of every person. That’s kind of an intimate thing for a new mom and baby that we didn’t get because we were always in front of people. It puts that extra set of eyes on you as a mom. (Maya)

In summary, parents expressed having to address unmet care gaps as hypervigilant, hyperresponsive care givers. Parents also had to accept care providers in their homes, impinging on their privacy. This burden of care resulted in stress and a sense of intrusion for parents and families.

## 5 DISCUSSION AND CONCLUSION

This is one of few studies describing the unstoried experiences of parents caring for children with rare NDDs. The narratives illuminate something new, different, and distinctive about parenting a child with a chronic illness and seem to contradict widely accepted social constructs surrounding parenting and caring for children with disabilities. Parents expressed feelings of normality and abnormality within a liminal space from what is familiar and unfamilar about the care experience.

The parent narratives resonated with impressions of silencing, being silenced and remaining silent when interfacing with systems and providers. Parents have been silent about their experiences because of discomfort from others and society with exposing struggle instead of valour and transcendence. Parents need to give voice to narratives of caring children with chronic illnesses, but they may be socially silenced within cultural narratives that depict them as a ‘good’ mother or parent.^11^, ^19^ Parents spoke of disillusionment within expectations from social constructs. There is a need to move from the cultural storyline of transformation with growth, betterment and transcendence to mitigate necessary medical and social supports for parents.^26^, ^43^ These findings enrich and expand current parenting constructs and care practices for families dealing with chronic disease and open the space for understanding.

The findings also relay how interacting with systems and providers has shaped, constrained and silenced parents’ voices about their experiences in caring for children with rare diseases. Parents described how health‐care and social care systems depend on parents to overcome care challenges as a valourized way of managing gaps in health care. This dominant dependence on parents may excuse the larger social and political contexts that need to be addressed beyond parents’ abilities^26^, ^44^, ^45^ and serve to create and sustain unrealistic expectations for parents. Our findings reveal the need to look beyond individual characteristics and family factors to larger policies and care delivery practices within social, political and health systems for children with complex needs.^1^, ^10^, ^44^, ^46^, ^47^ Current practice models provide disjointed fragmented care because of highly compartmentalized systems of medical management.^48^ Children see multiple care providers with expertise in highly specialized areas of care.^48^ Often, the child or family, despite or likely because of the numerous providers working with them, experience incomplete sharing of clinical information across programmes and systems, no pre‐planning for anticipated care disruptions and lack a shared plan of care.^49^, ^50^, ^51^ Our findings support an integrated care model with a case manager for the family, telehealth services for easier and timely access to care supports, adequate respite, emotional and peer support, and education and resources for medical and behavioural complexity.^1^, ^10^, ^44^, ^48^, ^52^, ^53^

As well, parents tried to overcome care challenges as unrecognized and silent therapists and care providers, often with little training or experience. Sousa coined the mother caregiver as a ‘warrior‐hero’^54^ ^(p220)^:

Mothers are expected to be nurturers as well as the external translators, advocates, and soldiers with expert, specialized knowledge in varied medical and nonmedical fields, including law, education, behavior analysis, pharmacology, sensory integration, motor therapies, and bureaucratic minutia.^54^ ^(p. 239)^

This is consistent with the work of MacKean et al, who described caregivers surrendering their roles as parents while taking on professional roles and jeopardizing their relationships and interactions with their children.^55^ As well, Macvarish et al described cultural pressure on parents to be their child's expert, providing therapy and intervention to prevent further delays.^56^ These imposed pressures contribute to parental feelings of intrusion, anxiety and failure, while overlooking broader allocation of health‐care resources and supports.

These are unrealistic caregiving expectations requiring sizable consideration of effort, money and training. If parents fail to provide or secure services for their children, there is a shift from hero status to blaming parents for not doing enough for their children and potentially worsening or exacerbating the disability.^57^ Parents may remain silent with care providers if they cannot transcend feeling overwhelmed and overburdened with predictably unpredictable home situations.^26^

Silencing of parents also occurred when experiences were misunderstood, suppressed or not validated by HcPs. Stories of struggle, normality and abnormality, and familiar and unfamiliar dimensions of parenting children within the findings enrich and expand current parenting constructs and care practices for families managing chronic disease. Parents’ care experiences need to be voiced, validated and legitimized by HcPs to inform an understanding and develop appropriate health and social support policies and practices for families. This need for validation of the struggle within the parent experience has rarely been revealed in the literature. 17 Validating others’ experiences provides an openness to difference, unfamiliarity and a genuine questioning of one's own assumptions and beliefs.^35^ White in his research on adult caregivers expressed the need to break the silence on difficulties and challenges with the caregiving experience: ‘When we begin naming our experiences as real, we bring our everyday challenges out of the shadows and into full light to be noticed and appreciated and valued’.^58^ White goes further and says, it is not enough to just be aware of what caregivers are experiencing—HcPs must acknowledge and validate the care parents provide for their children^58^:

But after people walk and drive far away from our situations, we are still here—in it. Other people will not make meaning for us. They will not find value in our experiences. They don’t have to—yet. They don’t need to—yet. We have to—now. We must—now. Or we risk getting stuck in others’ stale misperceptions that deny the very parts of everyday realities that we must inject with value, especially when they appear unbearable. (Z. White, personal communication, September 5, 2017)

Parents also described huge responsibility and burden when navigating medical algorithmic pathways and determining steps in care. As well parents became silenced and felt isolated when their expertise regarding their child was not sought or included in health or social plans of care. Parents as caretakers are increasingly pushed to invisible perimeters of hegemonic discourses that speak the language of disconnection and detachment. Not all parental experiences fit the form and forming of normative disability.

With the multitude of providers, parents felt silenced when they were not considered as part of the plan of care. Parents described amplifying their voices to get needed supports and described this struggle using raw primordial language. Optimizing family functioning must include unsilencing family narratives and amplifying parent experiences as story‐worthy. Barnert et al^59^ and Thomson et al^13^ stated the health of the child is intertwined with the health of the family and cannot be measured outside of the sphere of family health—the family is the patient. Our findings support acknowledging the family as a central unit of care^60^, ^61^; assisting parents with navigating and coordinating the broader social and medical systems; supporting disease and family transitions; and linking providers across institutions, community sites, and sectors.^62^, ^63^, ^64^, ^65^

This study has expanded our understanding of parents’ perceptions of caring for children with rare NDDs. Given these interpretations, we have an opportunity to inform the present understanding of caring for children with chronic illnesses while generating new strategies and directions for supporting families. More interpretive studies could give voice to the parent perspective to improve quality of life, care practices, and public policies for families living with a rare disease.

## 6 STRENGTHS AND LIMITATIONS

This study focused on the lived experiences of parents of caring for children with rare NDDs. The researchers interviewed parent participants and did not submit transcripts to participants for member checking, as is congruent with hermeneutic phenomenology.^66^ The researchers, however, did present the findings to stakeholders (including parents within local rare disease groups and HcPs), who acknowledged that the study outcomes aligned with their lived experiences. More research in this area will expand on parents’ experiences of caring for children with rare diseases.

## CONFLICT OF INTEREST

There are no conflicts of interest to declare.

## Data Availability

The data that support the findings of this study are available on request from the corresponding author. The data are not publicly available due to privacy or ethical restrictions.
